# Nutritional value, phytochemical composition, and antioxidant potential of Iranian fenugreeks for food applications

**DOI:** 10.1038/s41598-024-71949-4

**Published:** 2024-09-10

**Authors:** Ziba Bakhtiar, Mohammadreza Hassandokht, Mohammad Reza Naghavi, Mohammad Hossein Mirjalili

**Affiliations:** 1 Department of Horticultural Sciences, College of Agriculture and Natural Resources, University of Tehran, Karaj, Iran; 2 Division of Biotechnology, Department of Agronomy and Plant Breeding, College of Agriculture and Natural Resources, University of Tehran, Karaj, Iran; 3 Department of Agriculture, Medicinal Plants and Drugs Research Institute, Shahid Beheshti University, Tehran, 1983969411 Iran

**Keywords:** Antioxidant activity, Elements, Fenugreek, Metabolite, Vitamin, Volatile composition, Chemical biology, Plant sciences

## Abstract

Fenugreeks (*Trigonella* L. spp.), are well-known herbs belonging to the family Fabaceae, whose fresh and dried leaves have nutritional and medicinal value. In the present study, the content of phytochemical traits (essential oil, diosgenin, trigonelline, total phenol, total flavonoid, total saponins, and total tannins), bitterness value, pigments (chlorophyll, carotenoid, β-carotene, and anthocyanin), vitamins (group B vitamins and ascorbic acid), minerals, and antioxidant activity of thirty cultivated populations belonging to ten *Trigonella* species were evaluated. The species and populations were significantly different in all studied parameters. A significant positive and negative correlation (*p* < 0.05) was also observed between the studied parameters. In total, *T. teheranica*, *T. elliptica*, and *T. foenum-graecum* were distinguished as superior species. The results showed that fenugreeks leaves can be considered as a valuable source of food and phytochemical compounds. The obtained data can be help to expand the inventory of wild and cultivated *Trigonella* species for further exploitation of rich chemotypes in the new foods and specific applications.

## Introduction

The use of plants for nutrition and treatment has been at the same time as the history of human life. Most of the current foods and medicinal plants preparations have also been originated from the ancient^1,2^. In the last decade, there have been significant changes in global food consumption patterns, mainly due to population growth and economic development.

Food substitution for healthier nutrition such as meat replacement with protein-rich plant products is an emerging dietary trend that may change the future of food systems and environments around the world. Therefore, it is necessary to develop products that are beneficial for both human health and the environment^3^. Plant foods are related closely to the cultural, social, and economic aspects of human societies both in the past and present^4^. Wild plants have been used by the native people for a long time, and these plants still constitute a significant part of the global food basket^5^. For a long time, food improvement has been carried out for many agricultural crops including fruits, edible seeds, vegetables, and cereals, by selection and breeding of plant species, landraces, wild populations, accessions, and other vegetations^6–10^. Wild populations of plant species often form diverse chemotypes, and this diversity leads to differences in the scope of their medicinal and biological activities^11^.

Phytochemical studies of the plant species and populations led to the introduction of the best genotype and chemotype^12^ for further exploitation programs. Many studies have focused on the evaluation of the plant genetic resources in terms of phytochemical and nutritional value so far^7,13–15^.

Legumes have a special importance in the human diet because they have two or three times more protein content than cereals^16^. Fabaceae members play a special role in the human diet, folk medicine, food and pharmaceutical industries, and animal health worldwide^17^. In addition, plants are important in the sustainability of agricultural systems due to the improvement of physical, chemical, and biological properties of the soil^18^. Fabaceae is one of the largest families of flowering plants, which includes 750 genera and 20,000 species and is currently spread all over the globe. Beans (*Phaseolus* spp.), peas (*Pisum sativum* L.), broad beans (*Vicia faba* L.), green peas (*Vigna radiata* (L.) R. Wilczek), and lentils (*Lens culinaris* Medik.) are the most important and well-known legumes in the world^18^. Sixty species of the family (0.3%) have nutritional consumption for humans^3,16,19^. The genus *Trigonella* is an important member of legumes and comprises 135 species that are distributed across the world^20^. Fenugreeks especially *T. foenum-graecum* L. are originated from the Mediterranean region but the plant is widely distributed and cultivated in Europe, Africa, Asia, and Australia^21^.

Twenty *Trigonella* species are wildly distributed in all regions of Iran, of which six species are endemic and grow in the northwest, south, and center of Iran. Due to its high nutritional value and pleasant flavor and aroma, fenugreek (*T. foenum-graecum*) leaves and seeds are used as vegetables (fresh and cooked) and spice^22^. Young leaves of *Trigonella* species, especially *T. foenum-graecum* have been used in ancient times for various purposes such as indigestion in Greece and Egypt. Fresh and dried fenugreek leaves and stems are used as food flavoring^23^ and other medicinal treatments *i.e.* antipyretics, anemia, and stomach ulcers, and wound healing^18^.

Fenugreek leaves contain bioactive substances such as diosgenin, alkaloid pyridine trigonelline, saponins, tannins, various vitamins including thiamin, riboflavin, and vitamin C, and micro- and macro-elements such as K, Fe, and Cu^24–27^. Anti-diabetic, anti-cancer, anti-fungal, and anti-bacterial effects, protection of the liver and stomach, increasing the immune system, treatment of neurological disorders, and the effects of reducing fat, sugar, and blood pressure of the plant have been recently reported^28–30^. Phytochemical and nutritional traits of *Trigonella* species and their populations have been broadly studied. Various groups of chemical compounds including steroids, alkaloids, flavonoids, as well as coumarin and nicotinic acid, proteins, lipids, amino acids, and fibers have been extracted, identified, and isolated from these plants so far^25,26,28,31–34^. The evaluation of the phytochemical content and nutritional compounds of the herbs of *Trigonella* wild species and their populations has been limited in Iran, and mostly has focused on cultivated species, *T. foenum-graceum*^29,35,36^. While fenugreek seeds have been extensively studied for their various bioactive compounds and health benefits, the leaves of the plant have received less attention in scientific research^37^.

Such studies on the evolution and domestication of fenugreek during the last decade have enabled us to make a much better assessment of the product value, the necessity of collecting wild germplasms, their improvement potential, and the nature and number of resources available for this task.

Therefore, according to the presence of important specialized metabolites, high amounts of nutritious compounds including vitamins and elements, and their reported biological activity, the aim of the present study was to measure phytochemical compounds and determine of nutraceutical content of thirty populations of the ten *Trigonella* species from Iran.

The present study can be used to produce effective compounds from fenugreeks as a valuable plant source in the medicinal and food industries. The adequate plant species and their populations can be also considered in the breeding and cultivation systems for further commercial exploitation.

## Materials and methods

### Chemicals and reagents

Analytical grade reagents of all chemicals used in this study were purchased from Sigma-Aldrich Co. (Buchs, Switzerland) and Merck (Darmstadt, Germany).

### Plant materials and growth conditions

The seed samples of thirty populations belonging to 10 species of *Trigonella* L. collected across different geographical regions of Iran (Table 1) were prepared from Iranian Biological Resource Center (IBRC) and were sown at the Horticultural Research Station at University of Tehran, Mohammadshar, Karaj, Iran (N35° 46′, E50° 55′ at an altitude of 1320 m) in May 2021. The experiment was laid out in randomized complete block design (RCBD) with three replications. The cultivation site experienced a semi-arid climate with an average annual temperature of 14 ℃. During the growing season, the average minimum and maximum temperature ranges between 12 and 42 ℃. Precipitation during this period averaged 4.7 mm, with a relative humidity of 36.5%. The soil of the research site had a salinity of 0.71 ds/m, pH of 7.6, organic matter of 0.84%, lime of 5.60%, phosphorus (P) of 21.5 mg/kg, and potassium (K) of 340 mg/kg. The texture of the soil was loamy. Voucher specimens have been deposited for all studied populations in Herbarium of College of Agriculture and Natural Resources (Herbarium Instituti Agronomici Keredjensis) (HIAK), University of Tehran, Karaj, Iran.

**Table 1:** Geographic location of thirty studied populations of the ten *Trigonella* species from Iran.

No	Cultivated species	Code	Collection site	Voucher number	Geographical and environmental parameters of the plant origin				
					Latitude (N) Longitude (E)	Altitude (m)	RH^a^ (%)	MAP^b^ (mm)	MAT^c^ (°C)
1	*Trigonella astroides*	TAS1	Qazvin-Kouhin	HIAK-6566	36°37′, 49°65′	1130	52.0	310.2	14.2
		TAS2	Khuzistan-Mollasani	HIAK-6567	31°58′, 48°88′	25	39.5	216.4	26.1
		TAS3	Ilam-Mehran	HIAK-6523	33°07′, 46°10′	136	38.2	557.4	17.3
2	*T. calliceras*	TCL1	Guilan-Astara	HIAK-6942	38°26′, 48°52′	− 22	82.7	1322.6	16.1
		TCL2	Guilan-Bandar Anzali	HIAK-6943	37°46′, 49°46′	− 26	84.2	1892.0	15.2
		TCL3	Mazandaran-Behshahr	HIAK-6944	36°71′, 53°55′	− 28	79.0	621.5	17.7
3	*T. coerulescens*	TCO1	Ardabil-Meshginshahr	HIAK-6945	38°40′, 47°66′	1400	70.0	289.1	9.3
		TCO2	East Azerbaijan-Tabriz	HIAK-6946	38°35′, 46°30′	1460	52.1	272.2	12.1
		TCO3	West Azerbaijan-Khoy	HIAK-6947	38°20′, 44°77′	1396	57.5	319.4	11.2
4	*T. elliptica*	TEP1	East Azerbaijan-Mianeh	HIAK-6529	37°42′, 47°72′	1100	49.5	320.0	11.5
		TEP2	Kermanshah	HIAK-6573	34°23′, 47°00′	1374	38.3	415.1	14.9
		TEP3	Kurdistan-Mariwan	HIAK-6574	35°52′, 46°17′	1320	45.4	437.9	14.3
5	*T. filipes*	TFP1	Ilam-Salehabad	HIAK-6531	33°29′, 46°11′	136	37.2	550.7	17.4
		TFP2	Kermanshah-Qasr e Shirin	HIAK-6575	34°51′, 45°57′	333	36.8	395.6	15.1
		TFP3	Lorestan-Saravand	HIAK-6530	33°29′, 49°04′	450	42.0	486.0	16.9
6	*T. foenum-graecum*	TFG1	Hormozgan-Minab	HIAK-6513	27°15′, 57°07′	16	65.5	168.9	27.2
		TFG2	Isfahan-Ardestan	HIAK-6495	33°20′, 52°25′	1207	32.0	134.9	16.3
		TFG3	Razavi Khorasan-Mashhad	HIAK-6512	36°20′, 59°35′	1065	48.4	248.1	14.1
7	*T. spruneriana*	TSP1	Kohgiluyeh va BoyerAhmad	HIAK-6583	30°57′, 51°16′	1810	44.8	783.4	14.6
		TSP2	Fars-Dehbozorgi	HIAK-6582	29°63′, 52°51′	1500	35.3	329.3	18.0
		TSP3	Zanjan-Tashvir	HIAK-6536	37°13′, 48°30′	1638	53.0	311.8	11.5
8	*T. stellata*	TST1	Sistan va Baluchestan-Qasregand	HIAK-6948	26°32′, 60°18′	500	28.1	98.2	18.6
		TST2	Bushehr-Borazjan	HIAK-6949	29°21′, 51°20′	80	59.9	254.0	24.8
		TST3	Kerman-Kahnuj	HIAK-6950	29°90′, 57°66′	702	28.0	138.3	17.0
9	*T. strangulata*	TSG1	Lorestan-Khorramabad	HIAK-6951	33°48′, 48°35′	1147	41.6	487.1	17.3
		TSG2	Kurdistan-Ghorveh	HIAK-6952	35°16′, 47°81′	1900	45.1	417.8	12.8
		TSG3	West Azerbaijan-Urmia	HIAK-6953	37°55′, 45°08′	1363	58.5	327.1	10.7
10	*T. teheranica*	TTH1	Alborz-Karaj	HIAK-6954	36°47′, 51°42	2500	47.5	248.6	15.4
		TTH2	Mazandaran-Chalus	HIAK-6955	34°40′, 51°00′	1120	77.2	752.6	17.4
		TTH3	Tehran-Oushan	HIAK-6956	35°93′, 51°52′	1917	35.4	228.8	17.3

^a^RH: relative humidity.

^b^MAP: mean annual precipitation.

^c^MAT: mean annual temperature.

### Declaration statement

The authors confirm that the necessary permissions to collect the samples have been obtained and also the present study complies with the IUCN Policy Statement on Research Involving Species at Risk of Extinction and the Convention on the Trade in Endangered Species of Wild Fauna and Flora.

### Essential oil isolation and analysis procedure

The aerial parts of cultivated samples were collected at flowering stage from the research site and were considered for the essential oil isolation. For instance, the aerial parts of each sample (100 g) were subjected to hydro-distillation for 6 h, using a Clevenger-type apparatus^38^.

The essential oils were analyzed using an Agilent 7890A gas chromatograph (Agilent Technologies, Palo Alto, CA, USA). In the GC–MS analysis, an Agilent 5975C gas chromatograph coupled to a mass spectrometer (Agilent Technologies, Palo Alto, CA, USA). Analytical conditions were: helium as carrier gas (flow rate, 1.1 ml/min) with ionization voltage of 70 eV, injector temperature 250 ℃, detector temperature 300 ℃, split ratio (1:50), oven temperature program: 60–250 ℃ at the rate of 4 ℃/min and then held for 5 min. Mass spectra were recorded in the range from 50 to 550 *m/z*. The analysis was carried out on fused silica capillary DB-5 column (30.0 m × 0.25 mm, 0.25 μm film thickness) coupled with a TRACE mass (Manchester, UK). To identify the constituents of the essential oils, their mass spectra were compared with those of authentic standards from the internal reference mass spectra library^39^. From the GC data, the retention indices of constituents were calculated against those of *n*-alkanes (C_6_ to C_24_) and the oil on a DB-5 column under the same chromatographic conditions.

### Extraction and HPLC–PDA determination of diosgenin and trigonelline

Diosgenin extraction was carried out as described by Aminkar et al.^36^ with slight modification. For instance, the leaves of each sample (1 g) were added into a tube and 20 ml of 96% ethanol and then sonicated (Elma, S120H, Germany) for 30 min at room temperature. Then, 20 ml sulfuric acid (2N) was added and hydrolyzed under reflux conditions at 100 ℃ for 2 h. The suspension was centrifuged (centrifuge Rotanta 460r, Hettich, Germany) at 4400 rpm for 5 min. The mixture was partitioned with *n*-hexane. The *n*-hexane phase was dried under reduced pressure in a rotary evaporator (Heidolph Instruments GmbH, Schwabach Germany) at 35 ℃. Dried extract was solved in 3 ml acetonitrile and passed through the filter (0.22 μm).

Trigonelline extraction was performed as described previously^40^ with some modifications. Briefly, the leaves of the studied samples (1 g) were added into a tube with 5 ml of acetonitrile and then placed in a bath ultrasonic for 15 min at room temperature. Then, 20 ml of phosphoric acid (5N) and 20 ml of methanol were added and sonicated for 20 min. The mixture was concentrated in a rotary at 35 ℃ for reduce. Finally, the dry extract was dissolved at 3 ml acetonitrile and passed through the filter (0.22 μm).

Employing HPLC equipped with a diode-array detector with a C_8_ column (50 × 2 mm, 3 μm) and a UV detector (Waters 2487), the analysis was carried out. The following gradient system was used with acetonitrile/ water (90:10 v/v). The flow was maintained at 0.5 ml/min and column temperature at 25 ℃; sample injection was 20 μl. Absorbance was recorded at 210 and 263 nm for diosgenin and trigonelline, respectively.

### Extraction and determination of total saponin and total tannin content

Total saponin content (TSC) of the studied populations was determined as per the reported procedure^41^. One gram of powdered leaves of each sample was extracted using a microwave-assisted extraction method under 3 min irradiation time, 572 Watt microwave power, 64% ethanol concentration, and 1:10 g/ml solid-to-solvent ratio (1g leaf sample/10 ml ethanol). The samples were extracted in a microwave system (Milestone ETHOS UP, Italy). Accurately 100 μl extract was mixed with 400 ml methanol and 200 μl vanillin/ethanol (10:90 w/v). Then, 600 μl sulfuric acid (70%) was mixed and heated at 100 ℃ for 10 min. Absorbance was taken against the reagent blank (methanol) at 544 nm using a spectrophotometer (Bio-Tek Instruments, Inc., USA). A standard curve was calculated using diosgenin solution in the range 100‒500 mg/ml). Total saponin content was calculated as follows: [The volume of extraction solvent (ml) × The concentration measured from diosgenin standard curve (mg/ml)]/The dry weight of the sample (g).

Total tannin content (TTC) was measured according to Abdouli et al.^24,42^ with slight modification. Concisely, powdered leaves of each sample (100 mg) were mixed with 5 ml of diethyl ether containing 1% acetic acid and the mixture was placed on a magnetic stirrer for 15 min. The mixture was then centrifuged at 2000 rpm for 10 min. The supernatant was discarded and re-extraction was performed with 5 ml of acetone (70%) and stirring for 1 h. Finally, the extract was centrifuged at 2000 rpm for 20 min.

The total phenol content (TPC) in the extract was determined based on the Folin-Ciocalteu method and then 2 ml of diluted acetone extract was mixed with 100 mg of polyethylene glycol 4000 (for deposition Tannins). Total tannins content was calculated as the difference in total phenols content before and after the treatment.

### Determination of bitterness values

According to the WHO method^43^ and Abdouli et al.^42^, the bitterness value was assessed by comparing the threshold concentration of aqueous leaf extract (minimum concentration that still tastes bitter) with a dilute solution of quinine hydrochloride. In summary, 50 mg quinine hydrochloride and 50 ml drinking-water were mixed. Afterward, 5 ml of the solution was diluted to 500 ml with drinking-water. The stock solution of quinine hydrochloride contains 0.01 mg/ml. For preparing of stoke solution, the leaves of each sample (1 g) were extracted with 1000 ml drinking-water. A test panel consisting of ten adult members (male and female) was assembled. Bitterness value of the test solutions resulted from calculating the average of the individual values. Bitterness value was determined by using the below formula^43^.

Bitterness value (unit/g) = [2000 × Quantity of quinine hydrochloride with the lowest bitter concentration (mg)]/[Concentration of the stock solution (mg/ml) × volume of stock solution with the lowest bitter concentration (ml)]. The bitterness value of the solution was expressed as units/g.

### Determination of pigments

Photosynthetic pigments (chlorophyll a, chlorophyll b, carotenoid, and anthocyanin) were determined according to Missaoui et al.^44^ with minor modifications. For instance, fresh leaf tissues (0.1 g) were ground in acetone (10 ml, 80%). The mixtures were then centrifuged at 3,500 rpm for 10 min and supernatants were collected to determine Chla, Chlb, and carotenoid by reading the absorbance at 663, 645, and 470 nm, respectively, using a spectrophotometer (Shimadzu double beam UV–Visible spectrophotometer-1800, Japan). Leaf pigment concentrations were quantified according to the formulae of Lichtenthaler and Wellburn^45^ and expressed in mg/g FW.

Chlorophyll a = (11.75 × A_663_ ‒ 2.35 × A_645_) × 10 /100.

Chlorophyll b = (18.61 × A_645_ ‒ 3.96 × A_663_) × 10 /100.

Carotenoid = ((1000 × A_470_) – (2.27 × Chla) – (81.40 × Chlb) /227) × 10 /100.

β-Carotene content was determined by Negi and Roy^46^ method. The absorbance was read at 440 nm. β-Carotene concentration (1‒20 μg/ml) was determined from the standard curve. Anthocyanin content was calculated with grinding leaf tissues in 20 ml methanol/water/hydrochloric acid (16:3:1 v/v). The mixture was kept at room temperature for 2 days in the dark, then and centrifuged for 10 min at 13,000 rpm. The absorbance was read at 530 and 653 nm using a spectrophotometer. Anthocyanin content was calculated as follows: Anthocyanin = A_530_‒(0.24 × A_653_) with an extinction coefficient of 26,900 l/mol/cm^47^.

### Measurement of vitamin content

Type of vitamin B (Thiamin, riboflavin, niacin, and pyridoxine) was also determined following AOAC method^48^.

Leaf powder (1 g) was used for the determination of vitamin B groups. For instance, thiamin was calculated with adding 65 ml hydrochloric acid (0.1 N) to the plant sample. The sample was heated at 100 ℃ for 30 min in a water bath followed by centrifugation for 5 min at 4400 rpm. For riboflavin quantification 65 ml acetic acid–water mixture (50:50 v/v) was added to the plant sample. The mixture was heated at 100 ℃ for 30 min and stirred for 10 min in the dark. The solution for niacin was 3 ml hydrochloric acid (5 N), 3 ml dichloromethane, and 64 ml water. For extraction of pyridoxine, the plant sample was mixed to 12 ml sodium acetate solution (0.05 M) and pH was adjusted to 5.5. Absorbance was taken at 531, 460, 410, and 291 nm using a spectrophotometer for thiamin, riboflavin, niacin, and pyridoxine, respectively. The amounts of the vitamins were calculated as follows.

$$Vita\min \left( {\mu g/100 g DW} \right) = \frac{Absorbance of sample}{{Absorbance of s\tan dard}} \times \frac{{Dry weight of sample \left( {\mu g} \right)}}{Dry weight of sample \left( g \right)} \times 100$$

Ascorbic acid was determined using AOAC method^49^. Briefly, powdered leaf samples (500 mg) were extracted with 5 ml of 3% metaphosphoric acid and centrifuged at 4400 rpm for 5 min. The supernatant was titrated against 2,6-dichlorophenolindophenol dye solution (0.25%) to faint pink color. The ascorbic acid content was determined as follows: Ascorbic acid (μg/100 g DW) = [(Concentration of the standard ascorbic acid (μg/ml) × Titre value of the sample (ml) × 10) /(Titre value of standard ascorbic acid (ml) × Volume of the sample used (ml) × Weight of the sample (μg)] × 100.

### Elemental analysis

The leaves of the studied samples were prepared for digestion according to the method of Başgel and Erdemoğlu^50^ and Kan et al.^51^ with modifications. The leaf samples (500 mg) were digested using a microwave system (CEM, Mars X-Press, USA) with 6 ml pure nitric acid (69%) and 1 ml hydrogen peroxide (35%) in closed vessels with constant temperature heating at 140 °C for 3 h, and heated for digestion (4 h, 160 °C). Determination of mineral content by inductively coupled plasma mass spectrometry (ICP‒MS) Agilent 7500A series (Agilent Technologies, Palo Alto, CA, USA). Experimental conditions of the ICP-MS instrument were as follows; Rf power (W): 1100; Gas flow rate (l/min): Plasma gas: 15; Carrier gas: 1; Makeup gas: 1; Aux gas: 1; Spray chamber temperature: 2 °C; Torch: Quartz; Auto sampler: CETAC ASX-520; Read time (s): 30; Delay time (s): 60; Wash time (s): 15. Measurements of mineral content were checked using the certified values of related minerals in the reference samples received from the National Institute of Standards and Technology (NIST; Gaithersburg, MD, USA).

### Determination of total phenol and total flavonoid content and antioxidant activity

TPC of the studied samples was determined by the method of Singleton et al.^52^ with Folin-Ciocalteu’s reagent using gallic acid as the standard. Absorbance was measured at 765 nm against methanol as a blank. The total flavonoid content (TFC) was determined using the method of Chang et al.^53^ with aluminum chloride using rutin as the standard. Absorbance was determined at 510 nm versus prepared water blank.

The 1,1-diphenyl-2-picrylhydrazyl (DPPH) scavenging activity and ferric reducing-antioxidant assay (FRAP) methods were tested in the present study due to the fact that antioxidants are classified into two categories as water-soluble and fat-soluble antioxidants. These methods are high sensitivity and accuracy, fast, cheap, well established and popular for measuring the antioxidant properties of crude extracts or purified compounds from plants.

The DPPH was determined based on the method of Akhlaghi and Najafpour-Darzi^29^. The absorbance values were recorded at 515 nm. The inhibition percentage of anti-oxidative activity was determined using the equation: DPPH clearance = A_control_ – A_sample_)/A_control_ × 100%. The DPPH radical scavenging activity of butylated hydroxytoluene (BHT) was also assayed for comparison. The concentration providing 50% inhibition (IC_50_) was calculated using a calibration curve in the linear range by plotting the extract concentration vs the corresponding scavenging effect.

The FRAP solutions were prepared as described previously^54^. Ascorbic acid was used as the standard curve. The standard curve was constructed using iron (II) sulfate (FeSO_4_) solution. The absorbance of the mixture was then read at 593 nm using a spectrophotometer.

### Statistical analysis

All the analyses were run in three replicates. A one-way analysis of variance (ANOVA) was computed using the SPSS 25 software (SPSS Inc. Chicago, USA). The comparison between the data was evaluated using Duncan’s test, considering *p* < 0.05 and expressed as mean ± standard deviations (SD). The heat map and the plots were created by R statistical software (4.4.1), using the pheatmap, RColorBrewer, Viridis, ggplot2, tidyr, and corrplot packages. Canonical correspondence analysis (CCA) was evaluated using PAST software (4.03).

## Results and discussion

### Essential oil content and composition

The results showed that there is a significant difference (*p* < 0.05) between the species and populations in terms of content (% w/w) and identified components (%) of the oils. The content of the essential oils was in the range of 0.11 to 0.30% (Table 2). TEP2, TCL3, and TST1 had the highest essential oil content (0.30%), while the lowest (0.11%) was obtained in TAS1, TFG1, and TST3. According to previous reports, *Trigonella* species have low essential oil content. Ahmadiani et al.^55^ have reported the content of the essential oil in *T. foenum-graecum* as 0.3%.

**Table 2:** Volatile compounds of the studied populations of *Trigonella* species identified by GC-MS analysis.

No	Components	CRI^a^	TAS1		TAS2		TAS3		TCL1	TCL2		TCL3		TCO1		TCO2		TCO3		TEP1		TEP2		TEP3		TFP1		TFP2		TFP3
1	2-Acetylfuran	909	31.44		28.43		30.55		24.82	28.15		24.22		37.32		36.26		40.54		38.51		42.42		46.43		30.18		28.79		31.46
2	*n*-Nonanal	1100	4.54		5.53		4.56		5.33	0.97		1.58		–		0.56		1.29		0.23		–		–		4.55		4.57		4.76
3	Tetrahydrolavandulol	1157	2.66		2.24		3.81		7.35	7.92		7.91		1.68		0.11		4.71		0.75		0.63		0.82		0.20		1.50		2.61
4	2-Decen-1-ol	1268	5.81		6.00		5.52		5.03	6.01		5.98		8.43		7.01		11.81		0.45		5.56		5.78		7.05		8.02		6.83
5	1-Tetradecene	1288	1.00		1.12		1.16		–	–		–		1.07		–		1.37		0.87		2.15		4.46		1.07		1.08		1.00
6	Caryophyllen oxide	1582	0.30		0.56		0.29		3.01	2.32		–		–		–		–		–		–		–		0.29		0.35		1.70
7	Tetradecanal	1611	1.90		1.70		2.00		–	1.85		4.00		–		–		0.54		–		–		–		1.79		1.46		1.93
8	*allo*-Aromadendrene epoxide	1639	7.43		7.55		7.76		1.65	5.82		5.75		6.67		5.84		6.45		9.54		8.18		9.56		6.76		7.54		5.82
9	Hexadecyl acetate	2003	9.28		9.34		8.25		9.20	6.29		6.77		7.02		7.14		6.33		18.24		10.07		12.15		9.34		8.35		7.44
10	Octadecanol acetate	2209	34.61		36.61		34.64		42.35	39.29		42.31		37.45		41.24		26.32		29.61		28.77		18.68		36.57		37.42		35.09
	Oxygenated monoterpenes		2.66		2.24		3.81		7.35	7.92		7.91		1.68		0.11		4.71		0.75		0.63		0.82		0.20		1.50		2.61
	Oxygenated sesquiterpenes		7.73		8.11		8.05		4.66	8.14		5.75		6.67		5.84		6.45		9.54		8.18		9.56		7.05		7.89		7.52
	Others		88.58		88.73		86.68		86.73	82.56		84.86		91.29		92.21		88.2		87.91		88.97		87.50		90.55		89.69		88.51
	Total identified		98.97		99.08		98.54		98.74	98.62		98.52		99.64		98.16		99.36		98.20		97.78		97.88		97.80		99.08		98.64
	Oil yield (% w/w)		0.11		0.12		0.21		0.30	0.12		0.17		0.20		0.21		0.29		0.23		0.30		0.27		0.16		0.21		0.18

No	Components	CRI^a^		TFG1		TFG2		TFG3	TSP1	TSP2	TSP3		TST1		TST2		TST3		TSG1		TSG2		TSG3		TTH1		TTH2		TTH3	
1	2-Acetylfuran	909		31.63		33.76		32.51	22.52	29.41	30.92		25.77		24.75		21.60		30.50		31.27		30.51		38.49		38.44		38.64	
2	*n*-Nonanal	1100		5.56		5.56		5.77	7.52	–	3.56		1.73		1.41		1.59		5.54		5.60		5.72		–		0.25		–	
3	Tetrahydrolavandulol	1157		3.82		3.76		3.68	0.76	–	0.98		7.28		7.19		7.95		3.90		3.79		3.85		1.24		2.28		1.45	
4	2-Decen-1-ol	1268		5.90		5.82		5.87	6.00	7.81	6.01		6.00		5.78		6.00		6.00		5.81		5.83		6.00		1.81		2.87	
5	1-Tetradecene	1288		1.00		1.00		1.03	1.12	1.02	1.09		–		–		–		1.14		1.10		1.00		1.10		1.00		1.00	
6	Caryophyllen oxide	1582		3.20		2.28		2.27	0.67	1.59	1.52		3.00		3.13		2.73		2.89		2.77		2.27		0.71		–		–	
7	Tetradecanal	1611		1.77		1.70		1.99	0.67	1.78	4.51		1.16		1.79		1.79		2.00		1.85		1.83		–		–		–	
8	*allo*-Aromadendrene epoxide	1639		4.55		4.45		4.54	8.58	7.18	8.59		5.34		5.37		5.78		4.65		4.67		5.73		2.78		5.57		3.58	
9	Hexadecyl acetate	2003		6.25		6.24		6.25	8.00	9.60	6.24		6.13		7.19		6.56		6.69		6.94		6.56		4.67		8.22		5.21	
10	Octadecanol acetate	2209		35.18		35.37		35.92	43.96	40.64	34.74		40.61		41.27		45.14		36.27		35.08		35.05		43.25		39.63		45.60	
	Oxygenated monoterpenes			3.82		3.76		3.68	0.76	–	0.98		7.28		7.19		7.95		3.90		3.79		3.85		1.24		2.28		1.45	
	Oxygenated sesquiterpenes			7.75		6.73		6.81	9.25	8.77	10.11		8.34		8.50		8.51		7.54		7.44		8.00		3.49		5.57		3.58	
	Others			87.29		89.45		89.34	89.79	90.26	87.07		81.4		82.19		82.68		88.14		87.65		86.5		93.51		89.35		93.32	
	Total identified			98.86		99.94		99.83	99.8	99.03	98.16		97.02		97.88		99.14		99.58		98.88		98.35		98.24		97.20		98.35	
	Oil yield (% w/w)			0.17		0.11		0.15	0.15	0.14	0.15		0.30		0.11		0.18		0.19		0.15		0.12		0.14		0.14		0.21	

For a detailed description of the plant populations code, cf. Table 1.

^a^CRI: calculated retention indices determined in the present work relative to n-alkanes C_6_–C_24_ on DB-5 column.

**Fig. 1:**
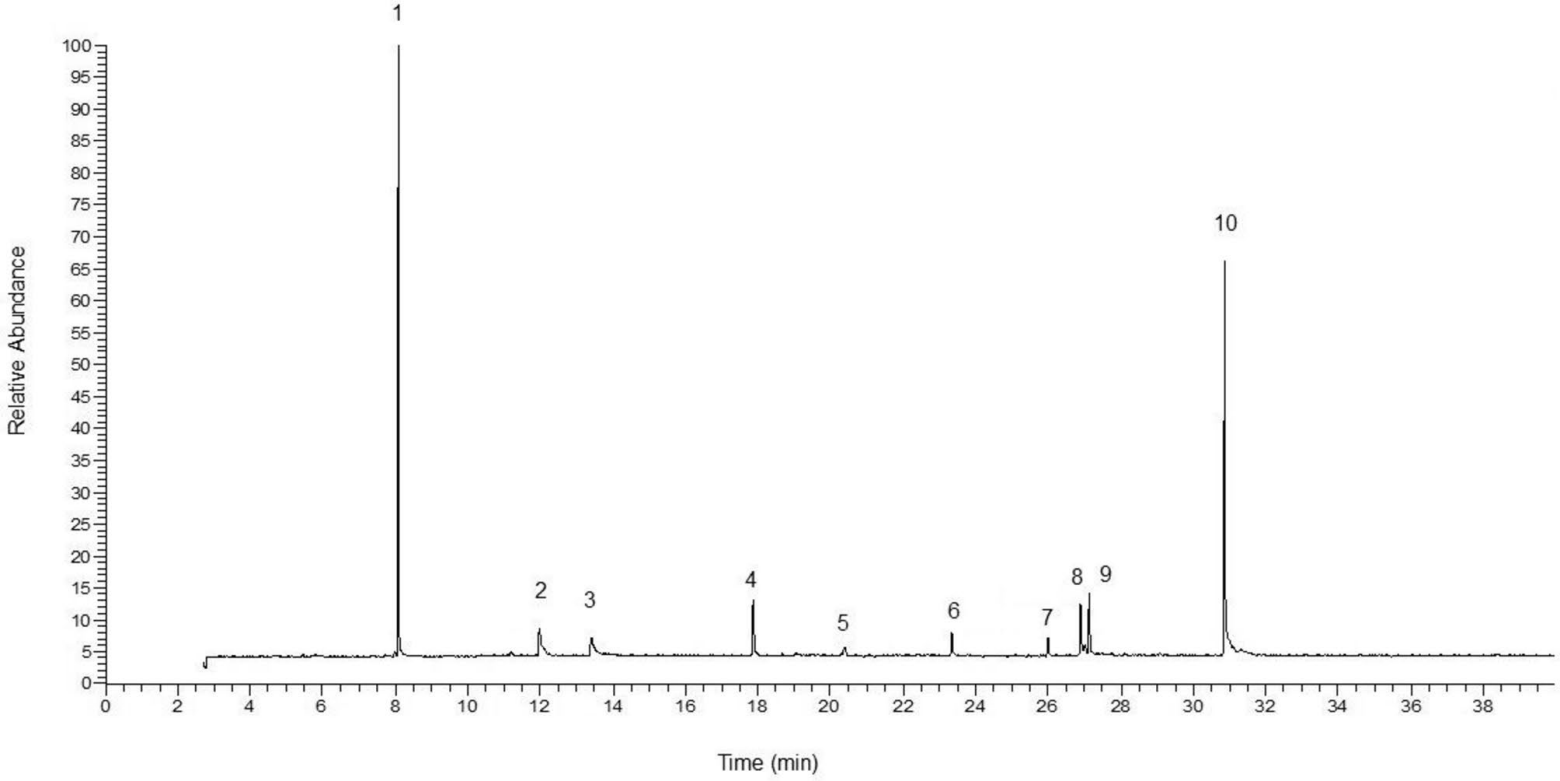
A typical GC–MS chromatogram of the studied essential oil sample. The numbers correspond to the compound ordered in Table 2.

Ten volatile compounds were identified in the studied samples, which comprised 97.02–99.94% of the total oil. The identified compounds were listed in Table 2 according to their calculated retention indices. 2-Acetylfuran (21.60‒46.43%) and octadecanol acetate (18.64–68.60%) were the major compounds of the studied essential oils. TEP2 and TEP3 had the highest percentage of 2-acetylfuran. The maximum content of octadecanol acetate was determined in TTH3 and TST3. Other identified compounds were hexadecyl acetate (4.67‒18.24%), 2-decen-1-ol (0.45‒11.81%), *allo*-aromadendrene epoxide (1.65‒9.56%), tetrahydrolavandole (0.00‒7.95%), and *n*-nonanal (0.00‒7.52%). TEP1 had the highest percentage of hexadecyl acetate, while the highest percentage of 2-decen-1-ol and *allo*-aromadendrene epoxide was obtained in TCO3 and TEP3, respectively. A typical GC-MS chromatogram of the studied essential oil sample is shown in Fig. 1.

**Fig. 2:**
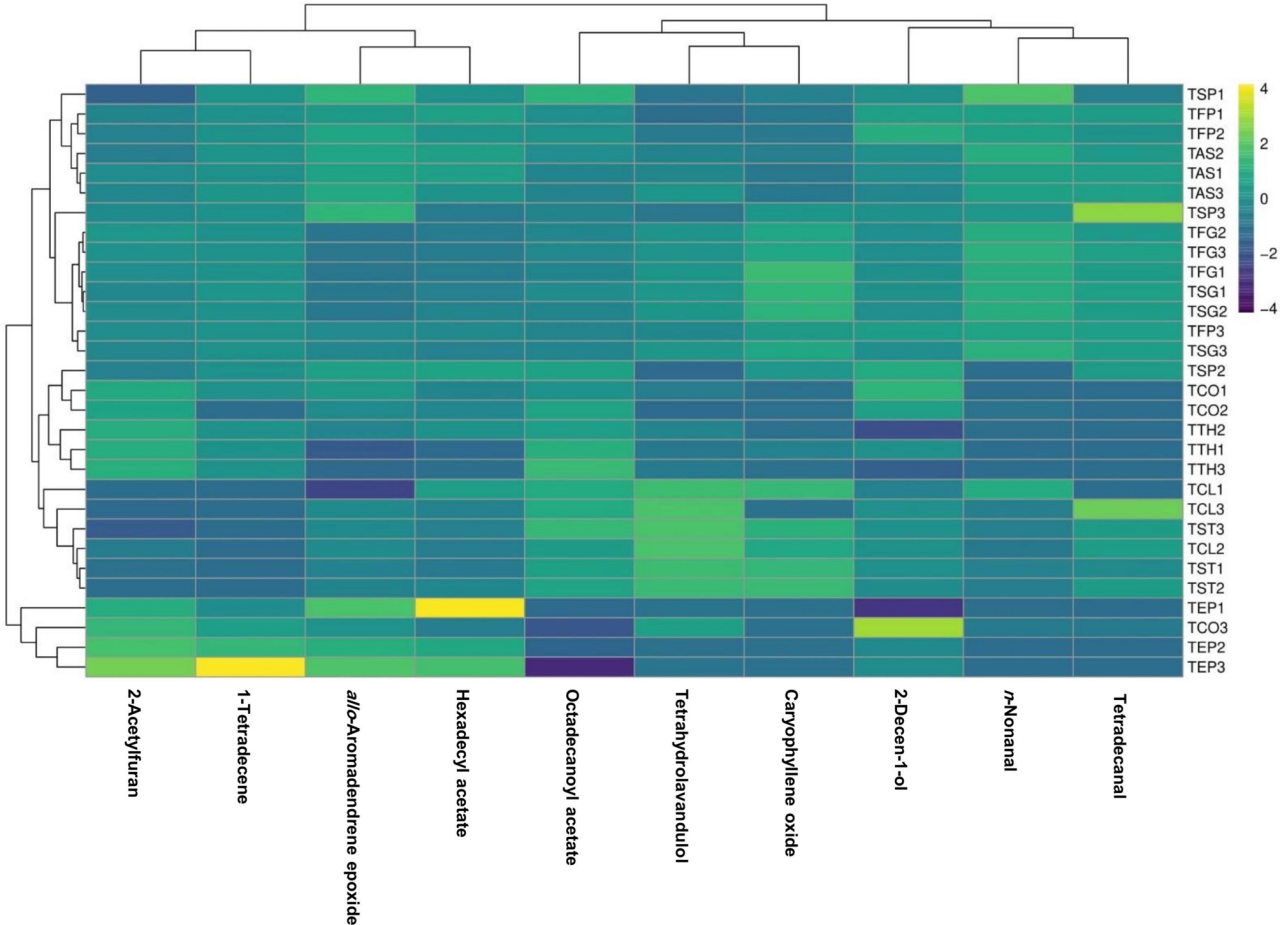
Heat map showing the essential oil profiles of thirty *Trigonella* populations. Mean values refer to colors from minimum displayed in dark blue to maximum represented with yellow. Created by R statistical software (version 4.4.1) using the pheatmap, RColorBrewer, and Viridis packages.

As can be seen in Table 2, oxygenated monoterpenes (0.00‒7.95%) and oxygenated sesquiterpenes (4.49‒10.11%) were the highest in oil. It has been reported that the plants of the legume family produce less mono-, sesqui-, and diterpenes than other plant families (Asteraceae and Lamiaceae)^56^. Heat map analysis based on the essential oil components is presented in Fig. 2. The studied fenugreeks populations were separated into two main groups. TEP1, TEP2, TEP3, and TCO3 were placed in one group and the other populations formed another group.

Riasat et al.^35^ have identified thirty-six compounds in the essential oil of *T. foenum graecum* leaves grown in Iran with the majority of 2*E*-hexenal (26.61%) and *n*-hexadecanoic (10.14%). Pentacosane (27.3%) has also been reported as the major composition in the essential oil of *T. disperma* Vassilcz. leaves from Iran^57^. Esmaeili et al.^58^ reported that dibutyl phthalate (10.3%), hexanal (9.5%), and nonanal (6.6%) were found to be the most common primary components in *T. monantha* C. A. Mey. subsp. *monantha* (synonym of: *Medicago monantha* (C.A.Mey.) Trautv.) from Iran. Also, Hajizadeh et al.^59^ reported germacrene-D (33.0%), bicyclogermacrene (26.0%), and viridiflorol (5.3%) as the main components in the essential oil of *T. torbatjamensis* Ranjbar aerial parts from Iran. In another investigation, *n*-hexadecanoic acid (20.84%), camphane (11.45%) and neo-menthol (5.05%) were found as the major essential oil compounds of *T. teheranica* (Bornm.) Grossh.^60^. Furthermore, ten components were totally characterized in the volatile oil of *T. foenum-graecum* from Iran with the majority of δ-cadinene (27.6%), α-cadinol (12.1%), γ-eudesmol (11.2%), and α-bisabolol (10.5%)^55^. Variation in the composition of essential oil content can be attributed to the genetic and different environmental conditions of their origins^61^.

2-Acetylfuran, is the most abundant flavoring compound in tamarind (*Tamarindus indica* L.), and its aroma in combination with α-terpineol, citral, and some rare pyrazines contributes to tamarind taste^62^. Octadecanol acetate, is also a chemical fraction of the sex pheromone of *Heliothis virescens* F., which prevents ovulation and is used in pest management^63^. Antibacterial^64^ and antifungal^65^ activity of *T. foenum-graecum* essential oil has also been reported. So, fenugreeks can be interestingly used to develop natural pesticides and fungicides to control pests and plant diseases. In the present study, TEP2, TEP3., and TCO3 are introduced as adequate populations rich in essential oil and 2-acetylfuran content. TST1 and TCL1 can also be considered for their high essential oil and octadecanol acetate content.

### Diosgenin and trigonelline content

A significant difference (*p* < 0.05) was observed among the studied species and populations in terms of diosgenin and trigonelline content. Variation of the diosgenin and trigonelline among thirty populations of the ten studied *Trigonella* species are presented in Table 3. The content of diosgenin in leaf extract was varied from 5.05 ± 0.01 to 24.52 ± 0.18 mg/g DW, while the content of trigonelline was in the range of 1.00 ± 0.00 to 4.21 ± 0.05 mg/g DW. The highest content of diosgenin was found in TFG2, TEP1, TFG1, and TFG3, while the maximum level of trigonelline was observed in TEP3, TFG3, and TFG2. Both TSG3 and TST3 had the lowest content of diosgenin and trigonelline.

**Table 3:** Variation of the diosgenin, trigonelline, total saponins, total tannins, and bitterness value among the populations of *Trigonella* species.

Populations	Diosgenin (mg/g DW)	Trigonelline (mg/g DW)	Total saponins (mg DE/g DW)	Total tannins (mg/100 g DW)	Bitterness value (units/g)
TAS1	7.21^d^ ± 0.01	1.29^c^ ± 0.02	27.32^de^ ± 0.07	269.66^ab^ ± 1.65	3.96^d^ × 10^3^ ± 0.08
TAS2	7.04^d^ ± 0.04	1.12^c^ ± 0.04	35.29^cde^ ± 0.26	197.43^c^ ± 1.23	5.58^c^ × 10^3^ ± 0.05
TAS3	6.16^d^ ± 0.02	1.08^c^ ± 0.06	26.88^de^ ± 0.16	290.56^ab^ ± 1.78	3.30^d^ × 10^3^ ± 0.02
TCL1	10.22^ cd^ ± 0.07	1.16^c^ ± 0.01	44.78^bcd^ ± 0.49	125.32^e^ ± 0.84	5.91^bc^ × 10^3^ ± 0.05
TCL2	6.09^d^ ± 0.01	1.94^bc^ ± 0.02	29.65^de^ ± 0.22	258.93^bc^ ± 0.59	5.25^c^ × 10^3^ ± 0.11
TCL3	10.75^ cd^ ± 0.02	2.42^b^ ± 0.01	56.44^a^ ± 0.80	145.32^de^ ± 0.78	6.79^b^ × 10^3^ ± 0.14
TCO1	18.33^a^ ± 0.02	3.12^a^ ± 0.03	60.17^a^ ± 0.71	154.28^cde^ ± 0.47	9.23^a^ × 10^3^ ± 0.21
TCO2	15.09^ab^ ± 0.10	2.96^ab^ ± 0.07	48.43^bcd^ ± 0.45	135.53^de^ ± 0.90	8.24^ab^ × 10^3^ ± 0.17
TCO3	12.87^c^ ± 0.12	3.04^ab^ ± 0.01	54.67^ab^ ± 0.17	156.43^cde^ ± 1.04	7.32^b^ × 10^3^ ± 0.12
TEP1	19.57^a^ ± 0.19	3.32^a^ ± 0.03	63.56^a^ ± 0.84	126.74^e^ ± 0.27	9.89^a^ × 10^3^ ± 0.09
TEP2	7.28^d^ ± 0.01	2.84^ab^ ± 0.01	38.18^ cd^ ± 0.42	191.53^ cd^ ± 0.66	5.62^c^ × 10^3^ ± 0.03
TEP3	12.44^c^ ± 0.06	4.21^a^ ± 0.05	55.42^a^ ± 0.28	129.76^e^ ± 0.43	7.87^ab^ × 10^3^ ± 0.08
TFP1	5.98^d^ ± 0.02	2.12^bc^ ± 0.03	54.64^ab^ ± 0.76	268.38^b^ ± 1.62	3.21^d^ × 10^3^ ± 0.10
TFP2	5.74^d^ ± 0.01	1.32^c^ ± 0.02	21.68^e^ ± 0.09	305.12^ab^ ± 1.46	3.53^d^ × 10^3^ ± 0.02
TFP3	6.18^d^ ± 0.03	1.24^c^ ± 0.05	25.54^de^ ± 0.14	311.90^a^ ± 1.12	4.75^ cd^ × 10^3^ ± 0.05
TFG1	18.62^a^ ± 0.15	3.33^a^ ± 0.06	66.13^a^ ± 0.35	98.21^e^ ± 0.55	9.43^a^ × 10^3^ ± 0.06
TFG2	24.52^a^ ± 0.18	4.01^a^ ± 0.01	66.37^a^ ± 0.59	94.32^e^ ± 0.73	10.32^a^ × 10^3^ ± 0.26
TFG3	16.24^a^ ± 0.14	4.18^a^ ± 0.05	62.25^a^ ± 0.56	100.45^e^ ± 0.85	9.64^a^ × 10^3^ ± 0.22
TSP1	7.68^d^ ± 0.02	3.22^a^ ± 0.05	41.36^bcd^ ± 0.95	170.49^cde^ ± 0.85	5.37^c^ × 10^3^ ± 0.12
TSP2	7.06^d^ ± 0.04	2.91^ab^ ± 0.01	29.61^de^ ± 0.08	198.28^c^ ± 1.15	5.11^c^ × 10^3^ ± 0.01
TSP3	6.25^d^ ± 0.03	2.53^b^ ± 0.04	20.43^e^ ± 0.11	214.37^bc^ ± 0.99	4.28^ cd^ × 10^3^ ± 0.08
TST1	5.82^d^ ± 0.05	1.12^c^ ± 0.04	19.85^e^ ± 0.08	367.81^a^ ± 1.35	4.41^ cd^ × 10^3^ ± 0.09
TST2	5.25^d^ ± 0.00	1.11^c^ ± 0.01	17.63^e^ ± 0.02	408.42^a^ ± 1.15	3.97^ cd^ × 10^3^ ± 0.01
TST3	5.12^d^ ± 0.01	1.02^c^ ± 0.09	18.52^e^ ± 0.10	347.48^a^ ± 1.00	4.45^ cd^ × 10^3^ ± 0.07
TSG1	7.02^d^ ± 0.04	3.96^a^ ± 0.03	37.07^ cd^ ± 0.21	199.40^c^ ± 1.34	5.57^c^ × 10^3^ ± 0.03
TSG2	5.30^d^ ± 0.02	1.24^c^ ± 0.07	18.45^e^ ± 0.18	317.96^a^ ± 1.57	2.68^d^ × 10^3^ ± 0.03
TSG3	5.05^d^ ± 0.01	1.00^c^ ± 0.00	17.52^e^ ± 0.11	408.43^a^ ± 1.37	1.50^d^ × 10^3^ ± 0.00
TTH1	11.65^c^ ± 0.05	2.28^b^ ± 0.01	58.61^a^ ± 0.34	165.32^cde^ ± 0.96	8.49^ab^ × 10^3^ ± 0.05
TTH2	11.84^c^ ± 0.11	1.15^c^ ± 0.05	54.22^ab^ ± 0.25	167.42^cde^ ± 1.28	8.12^ab^ × 10^3^ ± 0.11
TTH3	7.13^d^ ± 0.09	1.49^c^ ± 0.01	36.89^ cd^ ± 0.47	189.75^ cd^ ± 0.77	5.29^c^ × 10^3^ ± 0.06

Data expressed as mean ± standard deviation (SD) of three replicates. Different letters in column indicating statistically differences mean at *p* < 0.05 by Duncan’s multiple range test. For a detailed description of the plant populations code, cf. Table 1.

In a study on the leaves of four *Trigonella* species (*T. foenum-graecum*, *T. maritima* Delile ex Poir, *T. hamosa* L. and *T. stellata* Forssk.) from Egypt, the highest trigonelline content was found in the cultivated species. The compound was detected in all the leaf extract samples with the exception of *T. maritima*^37^.

Aminkar et al.^36^ were measured the content of diosgenin in the leaves of twenty-two populations of *T. foenum-graecum* from Iran. They reported the highest diosgenin level of 23.8 mg/g DW, which was similar to the results measured in the present study for the same species, but the content of diosgenin in other studied species was lower than that of *T. foenum-graecum* species. Dangi et al.^31^ reported the aerial parts of *T. foenum-graecum* contain 0.08 mg/g, while *T. caerulea* (L.) Ser. and *T. anguina* Delile had relatively higher diosgenin content (2.46 and 3.72 mg/g, respectively) than *T. foenum-graecum*. *Trigonella* can be a suitable and alternative source for the production of diosgenin. Therefore, finding the population of the plant with the highest potential can be of great importance for the production of diosgenin.

In a comparative study on diosgenin content in the aerial parts of the ten *Trigonella* species from Turkey, the highest diosgenin content (0.16 ± 0.00 mg/g) was reported in *T. cilicica* Hub.-Mor. Some of the studied fenugreeks including *T. kotschyi* Benth., *T. filipes* Boiss., and *T. strangulata* Boiss. lacked diosgenin in their aerial parts. They have also reported that *T. spruneriana* Boiss. contained 0.03 ± 0.00 mg/g diosgenin^26^ while, in the present study, the content of diosgenin in the populations of *T. filipes*, *T. spruneriana*, and *T. strangulata* was ranged from 5.05 ± 0.01 to 5.74 ± 0.02 mg/g DW (Table 3). In another study, diosgenin content was studied in the aerial parts of eleven varieties of *T. foenum-graecum* and the lowest and highest value were reported as 187.3 ± 0.5 and 466.9 ± 0.3 mg/100 g DW, respectively^34^. The results showed that the studied species and populations of *Trigonella* are different in terms of the content of specialized metabolites. The diversity of phytochemicals in plant populations and also species can indeed be influenced by a variety of intrinsic and extrinsic factors^12^. Intrinsic factors may include genetic diversity within the plant population, as different genotypes can produce different phytochemical profiles. Understanding the interplay between the factors can provide valuable insights into the ecological and evolutionary dynamics of plant populations, as well as their potential applications in agriculture, medicine, and other fields.

In the present study, we identified that *T. foenum-graecum*, *T. coerulescens* (M.Bieb.), and *T. elliptica* Boiss. have more content of diosgenin and trigonelline. By identifying high-productive species and potent populations within these wild fenugreek species, researchers can contribute to the development of sustainable and diverse sources of diosgenin and trigonelline. This not only broadens the availability of these valuable compounds but also offers opportunities for pharmaceutical industries to diversify their sources of raw materials for steroid drug synthesis. Furthermore, the exploration of other wild fenugreek species for their diosgenin and trigonelline content can contribute to the conservation and sustainable utilization of plant genetic resources. Understanding the potential of these wild species can also lead to the development of new cultivation strategies and breeding programs aimed at enhancing the production of diosgenin and trigonelline.

### Total saponin and tannin content and bitterness value

TSC, TTC, and bitterness value of the studied fenugreeks are presented in Table 3. A significant difference (*p* < 0.01 and *p* < 0.05) was observed between species and populations. The TSC of the leaf samples (17.52 ± 0.11‒66.37 ± 0.59 mg DE/g DW) varied greatly among the studied species and populations. The highest TSC content was in TFG1, TFG2, and TFG3. The lowest content was observed in TSG3 (Table 3).

The TTC was the highest in TSG3 (408.43 ± 1.37 mg/100 g DW) and TST2 (408.42 ± 1.15 mg/100 g DW). In other species it was found between 94.32 ± 0.77 to 367.81 ± 1.35 mg/100 g DW. TFG2 had the highest bitterness value in the leaf (10.32 ± 0.26 units × 10^3^/g), while the lowest bitterness value (1.50 ± 0.00 units × 10^3^/g) was obtained in TSG3.

Saponins can be found in the form of triterpenoid glycosides or steroids in a diverse array of plant-based foods, including legumes (0.0055–24.5%), cereals (0.04–12.47%), vegetables (0.001–4.7%), sugar beet leaves (5.8%), ginseng (1.4–5.58%), oil seeds (0.3–0.46%), yucca (10%), soapbark (9–19%), alfalfa herb (0.14–1.71%), and fenugreek (3.7%)^66,67^. However, toxic saponins are present in certain plant sources like leaves of some *Panicum*, *Sesbania*, and *Agave* species, seeds of *Bassia latifolia* (Fresen.) Asch. & Schweinf., fruits and flowers of *Nolina* species^68,69^. It has been interestingly indicated that safe limit for saponin consumption from various food sources, such as fenugreek is 2500 mg/kg of body weight^67,70^. Considering the obtained range of TSC in the studied fenugreeks, there will be no concern about their toxicity. Several studies have highlighted the positive effects of saponin consumption within safe limits, like the reduction of liver cholesterol and plasma in rabbits with a 1–1.2% saponin diet. Additionally, interactions between saponins and other antinutrients like tannins have been shown to decrease the individual toxicity of both compounds^71^.

In a study on optimizing the extraction conditions of TSC in *T. foenum-graecum* seeds from Malaysia, the highest reported content was 195.89 ± 1.07 mg DE/g DW^41^. This finding underscores the potential of fenugreek seeds as a rich source of saponins. Shawky et al.^37^ reported that saponin and saponin glycosides are abundantly found in the leaves of *Trigonella* genus. In another study, TSC and TTC in the leaves of *T. foenum-graecum* from Tunisia at the maturity stage have been reported as 0.33 and 7.02 g/100 g DW, respectively^24^. The lack of studies investigating the TTC and TSC, as well as the bitterness value of fenugreek leaves, highlights a significant gap in our understanding of this plant’s nutritional and sensory properties.

Previous studies have indicated that a range of factors, including the origin, plant species, environmental factors, and growing practices, influence the type and quantity of saponins present in food^72,73^. Saponin levels in crops can differ based on geographical location, plant species, and various stages of plant growth^67^. Considering the cultivation of fenugreek species and populations under the same agricultural conditions, the cause of variation obtained in the present study can be attributed to genetic factors.

The bitter taste of fenugreek leaves, attributed to compounds like saponins, can indeed pose a challenge for consumer acceptance and usage in daily diets^22,74^. Given the importance of sensory attributes in food acceptance, especially bitterness, it is crucial to explore debittering processes to enhance the palatability of fenugreek leaves^75^. By removing or reducing the compounds responsible for bitterness, such as saponins, the culinary appeal of fenugreek can be enhanced, potentially expanding its utilization in various food recipes.

Introducing less bitter populations of fenugreek species like *T. stellata*, *T. strangulata*, and *T. filipes* could be a promising approach to attract consumers who may have been deterred by the strong bitterness of traditional fenugreek varieties. By incorporating these less bitter alternatives into the food basket, consumers may discover new ways to enjoy the nutritional benefits of fenugreek without being put off by its bitter taste.

### Pigments data

**Fig. 3:**
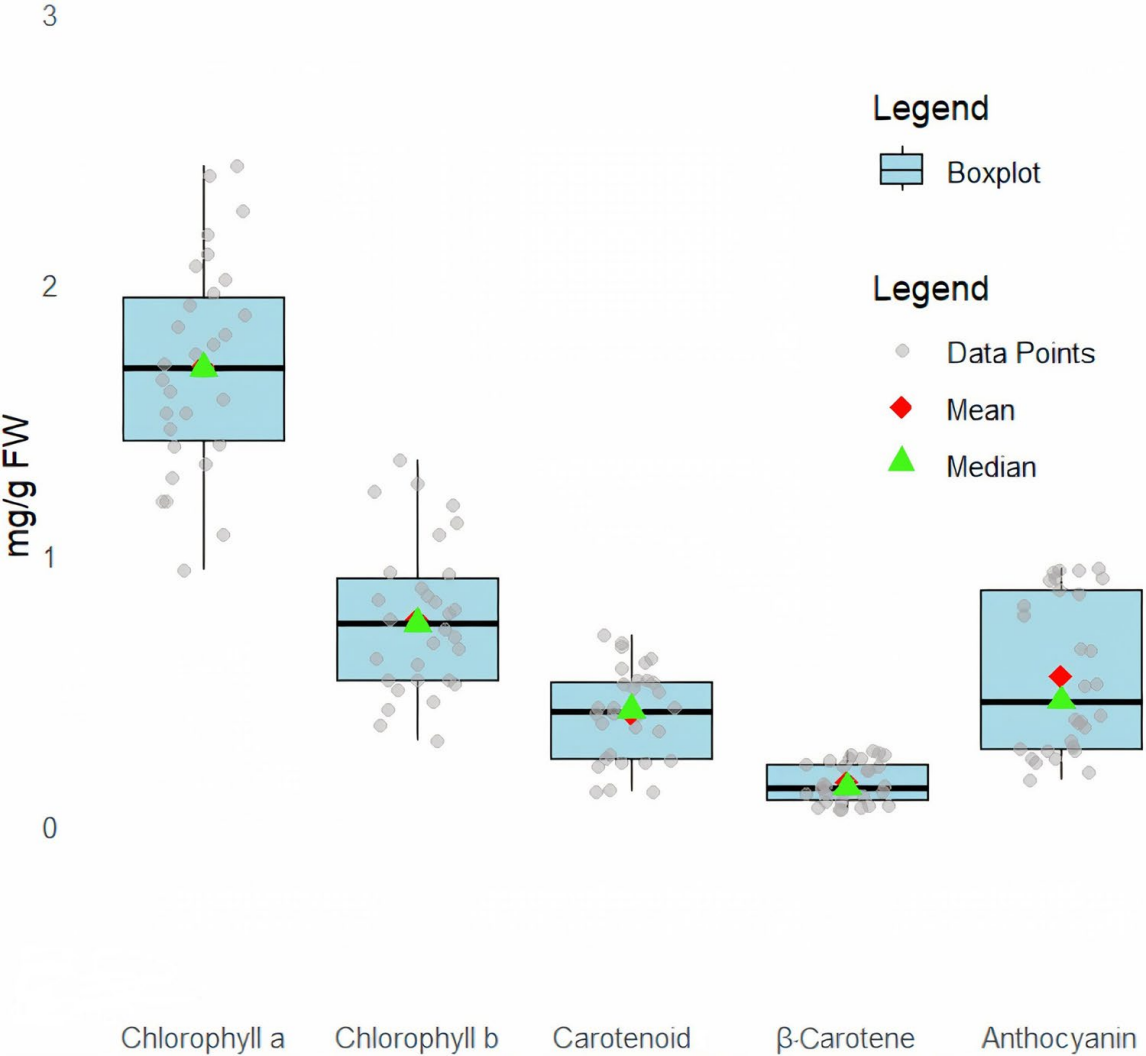
Values of different pigments among Iraninan *Trigonella* species.

The results showed that there is a significant difference (*p* < 0.05) among the studied fenugreeks in terms of leaf pigments content (chlorophyll a and b, carotenoid, β-carotene, and anthocyanin). The pigment content in the studied fenugreeks is shown in Fig. 3. The content of chlorophyll a in the studied fenugreeks was found in the range of 0.94 ± 0.03 to 2.43 ± 0.07 mg/g FW. TFG1 and TCO2 had the highest chlorophyll a content. The content of chlorophyll b in ranging 0.31 ± 0.04 to 1.34 ± 0.12 mg/g FW was lower than chlorophyll a. The highest and lowest chlorophyll b content was obtained in TCO1 and TAS1, respectively.

The carotenoid content in the leaves of the studied *Trigonella* species and populations was ranged from 0.12 ± 0.05 to 0.70 ± 0.14 mg/g FW, which showed the highest level in TCO1. β-Carotene content was in the range of 0.05 ± 0.01 to 0.27 ± 0.03 mg/g FW, and the highest value was obtained in TCO1, TCO2, and TCO3.

Hussain et al^76^ have reported that the leaf of *T. foenum-graecum* from India contained 128.1 ± 5.2 and 71.2 ± 2.5 mg/100 g of total chlorophyll and carotenoid content respectively. Joshi and Kulshrestha^77^ reported the β-carotene content of fenugreek leaves as 625 mg/100 g DW. In a study, β-carotene content in fenugreek leaves was obtained 28.1 mg/100 g DW^46^. In another study, β-carotene content of fresh *T. foenum-graecum* leaves was reported as 19 mg/100 g^78^. The anthocyanin content was found in the range of 0.16 ± 0.02 to 0.95 ± 0.01 mg/g FW and the highest value belonged to the TFP1. In many studies, chlorophyll, carotenoid, and anthocyanin contents of *T. foenum-graecum* were investigated and similar values were reported^79–82^. In a study, leaf chlorophyll a and b, and carotenoid content of *T. corniculata* Sibth. & Sm. were reported 1.36, 0.64, and 0.6 mg/g, respectively^33^.

The effect of genetic and climatic conditions such as light, temperature, and precipitation on the content of pigments has been extensively reported^83,84^. Since the pigments are natural antioxidants, as a result, the protective and therapeutic role of *Trigonella* seems to be different in the studied fenugreeks. This issue is very important in the food industry, especially the production of edible pigments, as well as the pharmaceutical industry. The findings regarding the high carotenoids, β-carotene and anthocyanin content in the populations of *T. coerulescens*, *T. foenum-graecum, and T. teheranica* species suggest that these species have potential for further exploitation in cultivation and breeding programs to meet the demands for food and pharmaceutical applications.

### Vitamin content

**Fig. 4:**
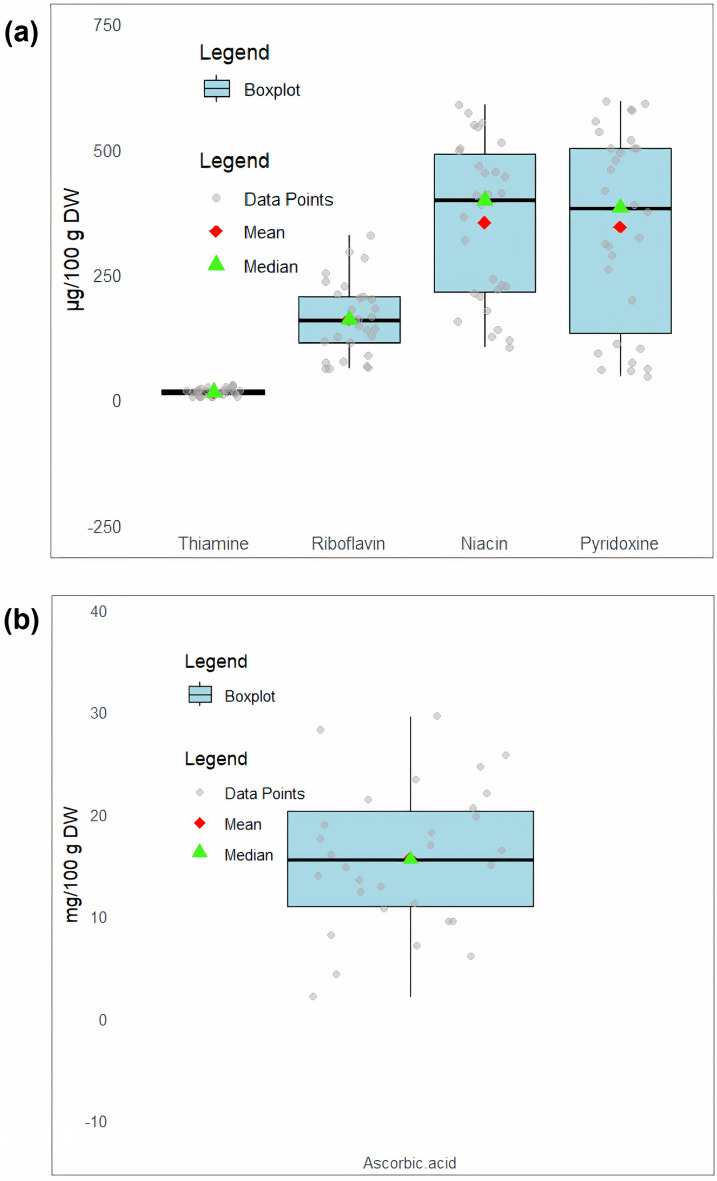
Values of different vitamins among Iraninan *Trigonella* species.

Vitamin content was significantly (*p* < 0.05) varied among the studied *Trigonella* species and populations. The level of vitamins (group B vitamins and ascorbic acid) studied in the plant samples are presented in Fig. 4a,b. Thiamin content was the lowest among group B vitamins. TFP1, TFP2, TFP3, TTH1, TTH2, and TTH3 had the highest thiamine content (19.43 ± 0.85‒29.43 μg/100 g DW). The lowest thiamine content was obtained in TCO1, TCO2, TCO3, TSP1, TSP2, and TSP3 (5.64 ± 0.27‒4.65 ± 0.23 μg/100 g DW). Riboflavin content varied from 61.13 ± 0.69 to 327.46 ± 1.56 µg/100 g DW. The highest riboflavin content was obtained in TFP1, TFG2, and TFG1, while the lowest content was found in TSG2 and TSG3. Niacin level was ranged from 105.15 ± 1.68 to 588.80 ± 1.24 µg/100 g DW. The highest niacin content was measured in the plant materials of TEP3, TFG1, TFG2, and TFG3, while the lowest level was observed in TSG3. The content of pyridoxine varied from 46.26 ± 1.53 to 595.21 ± 1.25 µg/100 g DW. Among the measured vitamins, ascorbic acid had the highest level that measured in the plant materials of the TCL2 population of *T. calliceras* Fisch. (29.55 ± 0.91 mg/100 g DW). TTH1, TTH2, TTH3 also had high values of ascorbic acid (24.48 ± 0.62‒28.45 ± 1.01 mg/100 g DW).

It has been reported that the leaves of *T. foenum-graecum* contain 40, 310, and 800 µg/100 g of thiamine, riboflavin, and niacin, respectively. The leaves of the species also contained 52 mg/100 g ascorbic acid^22,27^. In a study, the ascorbic acid content of *T. foenum-graecum* leaves from India was found to be 1047.4 mg/100 g DW^46^. Yadav and Sehgal^78^ stated that fresh *T. foenum-graecum* leaves contain about 220.97 mg of ascorbic acid per 100 g. According to Hussain et al^76^ total ascorbic acid of *T. foenum-graecum* leaves from India was determined as 51.4 ± 1.2 mg/100 g.

It is estimated that more than two billion people in the world, most of them in developing countries, are vitamin deficient. Vitamin deficiency leads to low quality of life and reduced economic productivity^85^. The present study showed that the leaf of the *Trigonella* species especially *T. foenum-graecum* has high vitamin content that can be used in different diets and the formulation of various food supplements.

### Macro- and microelements data

**Fig. 5:**
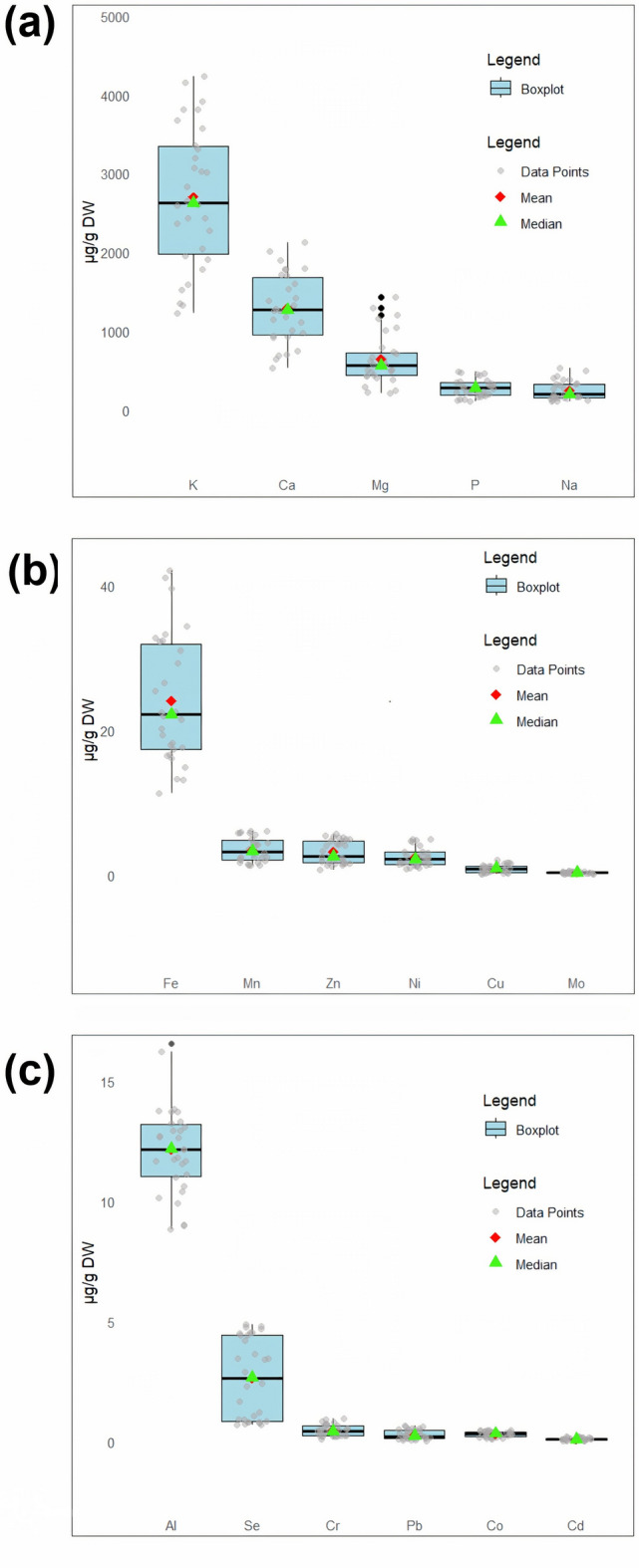
Box plots showing variation in levels of elements among Iraninan *Trigonella* species: (**a**) macroelements, (**b**) microelements, and (**c**) others.

There is a significant difference (*p* < 0.05) among the studied fenugreeks in terms of element content. Figure 5a–c shows the boxplot of macro- and microelements of the studied Iranian fenugreek species. The content of K was found in the range of 1222.34 ± 6.09 to 4233.82 ± 17.39 μg/g DW, of which the highest value was measured in TCL1. Another common macro-element was calcium (Ca), which was found in the range of 524.34 ± 8.29 to 2124.59 ± 9.74 μg/g DW. The plant materials of TFP2 contained the highest level of Ca. Other measured macro-elements were magnesium (Mg) (217.16 ± 4.80‒1431.43 ± 6.55 μg/g DW), P (114.52 ± 1.67‒482.96 ± 5.48 μg/g DW), and sodium (Na) (529.81 ± 3.64‒111.00 ± 1.41 μg/g DW). Among the micro-elements, iron (Fe) had the highest content (42.13 ± 1.17 µg/g DW) that measured in TCO1. Other elements in the leaves descending order included aluminum (Al), manganese (Mn), zinc (Zn), nickel (Ni), selenium (Se), copper (Cu), chromium (Cr), molybdenum (Mo), lead (Pb), cobalt (Co), and cadmium (Cd).

Evaluation of mineral element diversity (K, Na, Ca, Mg, and Fe) in the aerial parts of thirty-eight cultivars of *T. foenum-graecum* with the majority of K has been previously reported^72^. In a study, the value of nutritional elements in the aerial parts of *T. foenum graecum* and *T. corniculata*, showed that these species are excellent sources of Ca (10988 and 11364 μg/g DW, respectively), Mg (4501 and 4282 μg/g DW), K (17644 and 12260 μg/g DW), and P (3545 and 3933 μg/g DW). The content of Fe in the aerial parts (318 μg/g DW) of *T. corniculata* was also higher than that of *T. foenum-graecum* (293 μg/g DW)^32^. Gharneh and Davodalhosseini^86^ showed considerable differences of Ca (200.66–455.25), P (182–250), Mg (130.6–370.1), K (26.13–36.40) Na (27.77–59.40), Fe (8.10–30.23), Mn (0.10–0.87), Zn (1.04–4.13), and Cu (0.50–2.54) content (mg/100 g FW) among the leaves of seven *T. foenum-graecum* genotypes from Iran. They stated that the observed difference in nutrient composition of fenugreek genotypes is consistent with relatively high genetic diversity.

Uras Güngör et al.^26^ have measured the content of macro- and micro-elements in the aerial parts of the ten *Trigonella* species grown in Turkey. In their report, K content varied from 5225 to 13327 μg/g DW with the majority in *T. cilicica*. Ca content was in the range of 7466 to 13754 μg/g DW. The Fe level was varied from 35 to 285 μg/g DW in their plant materials studied. They have been reported the highest content of Ca and Fe in *T. spruneriana* and *T. smyrnaea* Boiss*.*, respectively^26^.

Macro- and microelement content in the leaf of the studied fenugreeks populations is largely an inherent feature, although they are also determined by climate and cultivation practices, which explains the significant differences in the results noted by various authors^26,32,86,87^.

In the present study, it was found that the leaves of the studied fenugreeks have a high content of macro- and micro-elements, which increases the nutritional value. The leaves of the studied species and populations were also a good source of Fe, so it can be recommended in the diet of individuals with iron deficiency. According to the maximum allowed level reported by the WHO^88^, the concentration of heavy metals was not higher in any of the studied samples. Soil factors such as pH, organic matter content, and mineral composition can indeed play a significant role in determining the trace element composition of plants^89^. Understanding the interactions between the properties and plant uptake of trace elements is important for managing the levels of the elements in crops and ensuring food safety. The present study showed that the leaves of the studied fenugreeks are a good source of nutrients that can play an important role in diets and prevention of various diseases.

### Total phenol content, total flavonoid content, and antioxidant properties

**Fig. 6:**
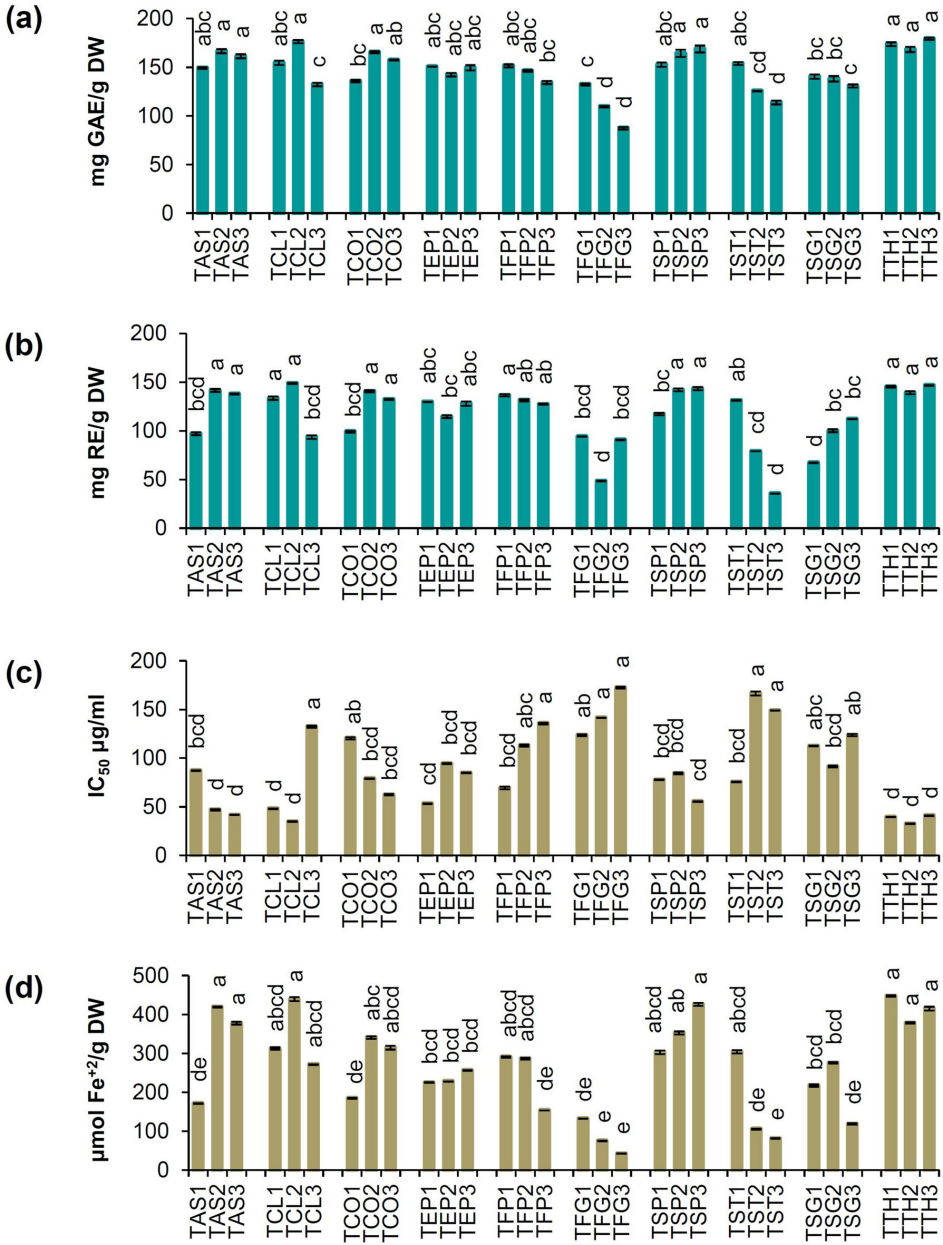
Total phenol (**a**) and flavonoids (**b**) content, and antioxidant activates (**c, d**) in the leaf of the studied populations of *Trigonella* species. n = 3, error bars represent standard deviation.

TPC, TFC, and antioxidant properties of the studied samples are shown in Fig. 6a–d. The TPC was ranged from 87.43 ± 1.23 to 179.10 ± 1.06 mg GAE/g DW. The highest content of TPC was measured in some populations of *T. teheranica* (TTH1, TTH2, TTH3) followed by TCL2.

Similar values (83.46 ± 0.24 to 139.88 ± 0.38 mg/g) have been reported for TPC in the ten *Trigonella* species from Turkey^26^. In the present study, the TFC was varied from 35.51 ± 0.07 to 148.72 ± 0.56 mg RE/g DW and the highest content was found in TCL2.

The lowest antioxidant activity by DPPH method belonged to TFG3 (IC_50_ = 172.42 ± 0.92 μg/ml), while the highest antioxidant activity (IC_50_ = 32.43 ± 0.38 μg/ml) was measured in TTH2. Antioxidant power by FRAP method was found in the range of 42.27 ± 0.08 to 447.49 ± 1.78 μmol Fe^+2^/g DW. The highest antioxidant power was obtained in TTH1, TTH2, TTH3, TSP3, and TCL2.

The Iranian *Trigonella* species and populations evaluated in this study showed significant variability in terms of TPC, TFC, and antioxidant activity. These results are similar to the findings of other researchers. For example, antioxidant activity in the leaves extract of *T. foenum-graecum* has been already reported as 44.89 μg/ml^29^. In another study, antioxidant activity of *T. arabica* Delile and *T. berythea* Boiss. has been reported as 12.50 ± 0.54 and 6.02 ± 0.44 µg/ml. TPC were also obtained as 885.34 ± 1.14 and 64.44 ± 1.44 mg GAE/g, and TFC were 93.97 ± 0.4 and 76.67 ± 1.1 mg RE/g, respectively^90^. Gupta and Prakash^91^ obtained the antioxidant activity of *T. foenum-graecum* as 27 mg/ml. A wide range of TPC, TFC, and antioxidant activity has been reported in *Trigonella* species so far^22,92^. Hussain et al^76^ reported the TPC and TFC of *T. foenum-graecum* leaves from India as 425.4 ± 10.6 and 205.1 ± 8.9 mg/100 g, respectively.

**Fig. 7:**
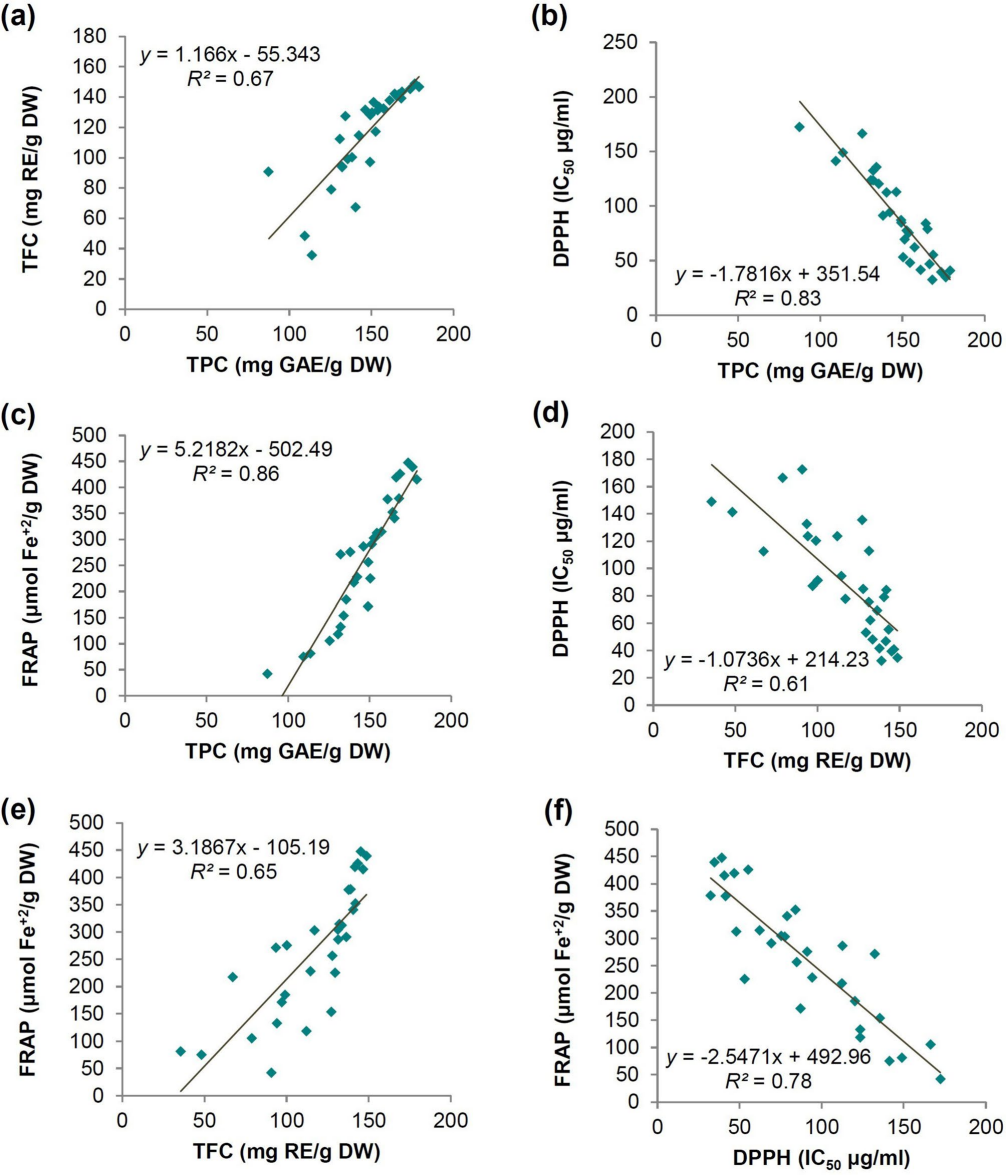
Linear correlation between total phenol and flavonoids content, and antioxidant properties (**a‒f**). (Significant level: 0.05, n = 30).

Our results revealed that the studied samples have a high TPC and TFC, which led to an increase in their antioxidant activity. Correlation analysis showed a significant relationship between TPC, TFC, and antioxidant properties of the studied *Trigonella* species and populations (Fig. 7a–f). A significant relationship was found between TPC and antioxidant properties by DPPH (*R2* = −0.83) and FRAP method (*R2* = 0.86). TFC also had a significant relationship with the antioxidant properties by the DPPH (*R2* = −0.61) and FRAP (*R2* = 0.65). Nowadays, most of the researches are focused on the use of new and safe antioxidants from plant sources^93^. Due to cancer incidence increasing globally, the importance of studying antioxidant compounds, especially in plant sources, has been clarified^94^. Alternatively, through targeted breeding efforts and selection processes, breeders can create new fenugreek cultivars that not only meet specific nutritional needs but also useful for special pharmaceutical purposes.

### Principal component analysis and correlations among traits

**Fig. 8:**
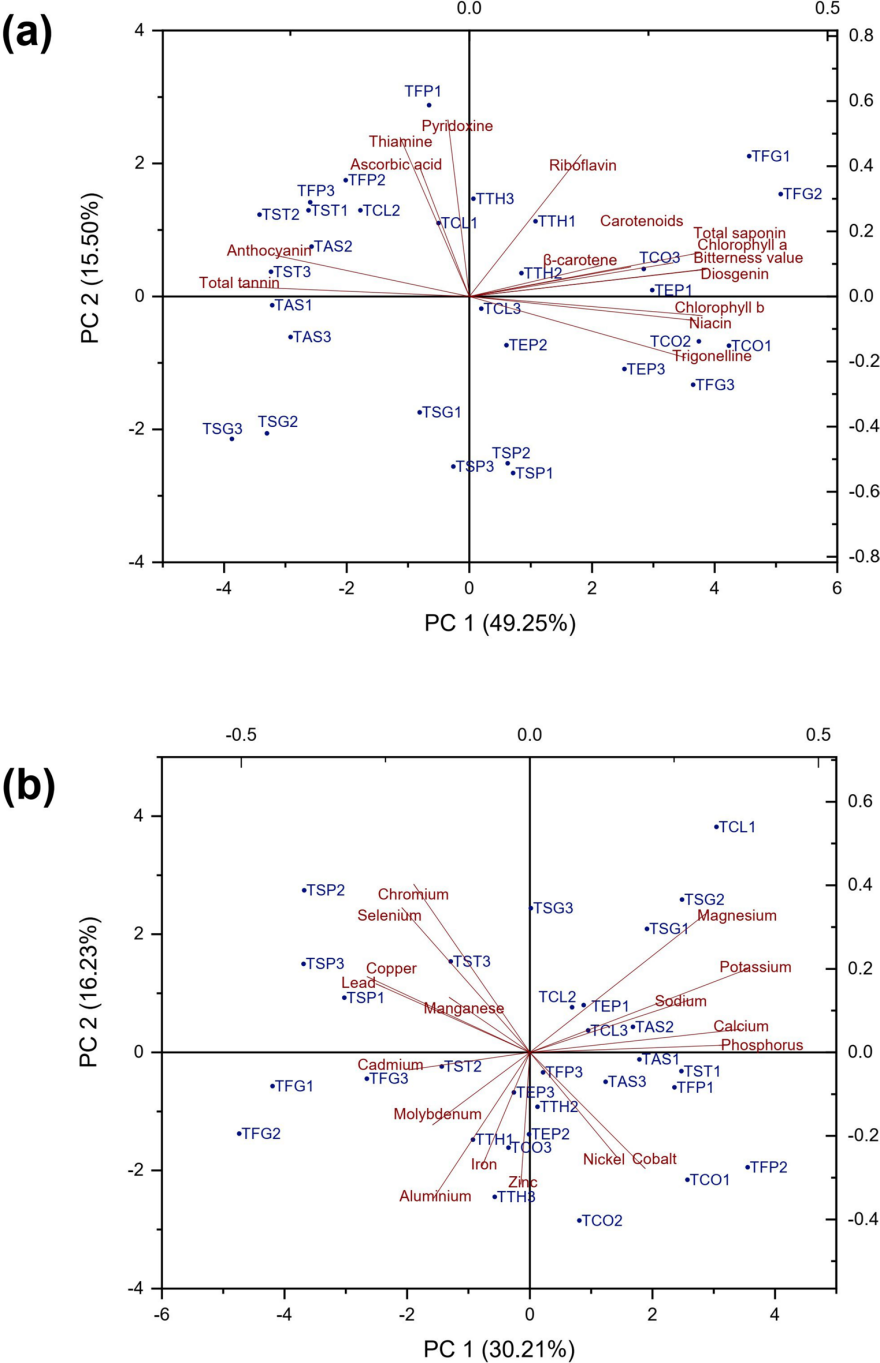
Principal component analysis (PCA) graph of quantified studied parameters: (**a**) specialized metabolites and (**b**) elements. For a detailed description of the plant populations code, cf. Table 1.

Biplot analysis was performed using PC1 and PC2, which accounted a total of 64.75 and 46.44% of the variance for phytochemical compounds and elements, respectively (Fig. 8). According to the biplot of phytochemical compounds, the populations were divided into four groups (Fig. 8a). TFG1, TFG2, TTH1, TTH2, TTH3, TCO3, and TEP1 characterized with high values in riboflavin, β-carotene, carotenoid, TSC, chlorophyll a, bitterness value, and diosgenin were placed in the first group, while TCO1, TCO2, TEP2, TEP3, TFG3, TCL3, TSP1, and TSP2 formed the second group that characterized by high value in chlorophyll b, niacin, and trigonelline. The third group including some populations of *T. strangulata* (TSG1, TSG2, TSG3), *T. astroides* Fisch. & C.A.Mey. (TAS1, TAS3), and *T. spruneriana* (TSP3) were not characterized by any phytochemical compounds studied. The highest content of total tannins, anthocyanin, ascorbic acid, thiamine, and pyridoxine was found in TAS2, TCL1, TCL2, TST1, TST2, TST3, TFP1, TFP2, and TFP3, which were placed them in the fourth group.

The studied samples were separated into four groups based on element content (Fig. 8b). TCL1, TCL2, TCL3, TSG1, TSG2, TSG3, TEP1, and TAS2 were formed the first group which were associated with high value in macro-elements. In addition, nine populations formed the second group, which were characterized by high content of Ni and Co. TFG1, TFG2, TFG3, TST2, TTH1, TTH3, TEP2, TEP3, and TCO3 were placed in the third group that characterized by Zn, Al, Fe, Mo, and Cd content. The fourth group including TSP1, TSP2, TSP3, and TST3 was related to high values in Cr, Se, Pb, Cu, and Mn.

**Fig. 9:**
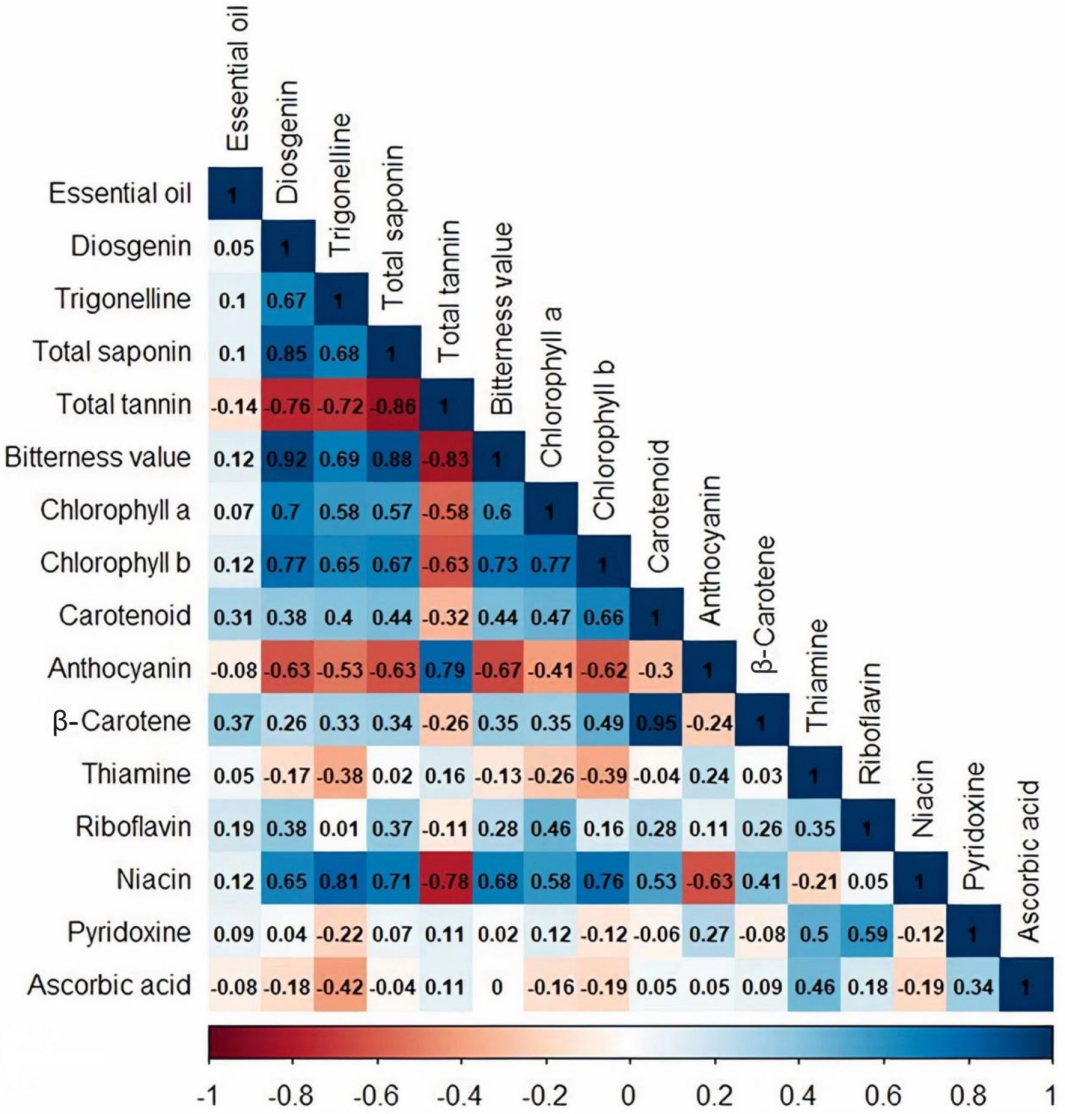
Correlation plot based on the specialized metabolites for the studied populations of *Trigonella* species (Significant level: 0.05).

Correlation analysis showed a significant positive and negative relationship (*p* < 0.05) among the studied phytochemical traits. The content of niacin, chlorophyll b, diosgenin, trigonelline, TTC, TSC, and bitterness value had the most significant relationship between the studied traits (Fig. 9). β-Carotene content had the highest positive and significant relationship with carotenoid (*r* = 0.95) followed by diosgenin content with bitterness value (*r* = 0.92). The strongest negative and significant relationship was observed between TTC and TSC (*r* = − 0.86) and bitterness value (*r* = − 0.83). As mentioned before, correlation analysis was also showed that the bitterness value of fenugreek leaves is due to saponin compounds including diosgenin^74^. A significant relationship between phytochemical traits in plants has been previously reported^95–97^. In addition to genetic, plant growth conditions play a key role in the value of compounds^11^.

### Canonical correspondence analysis

**Fig. 10:**
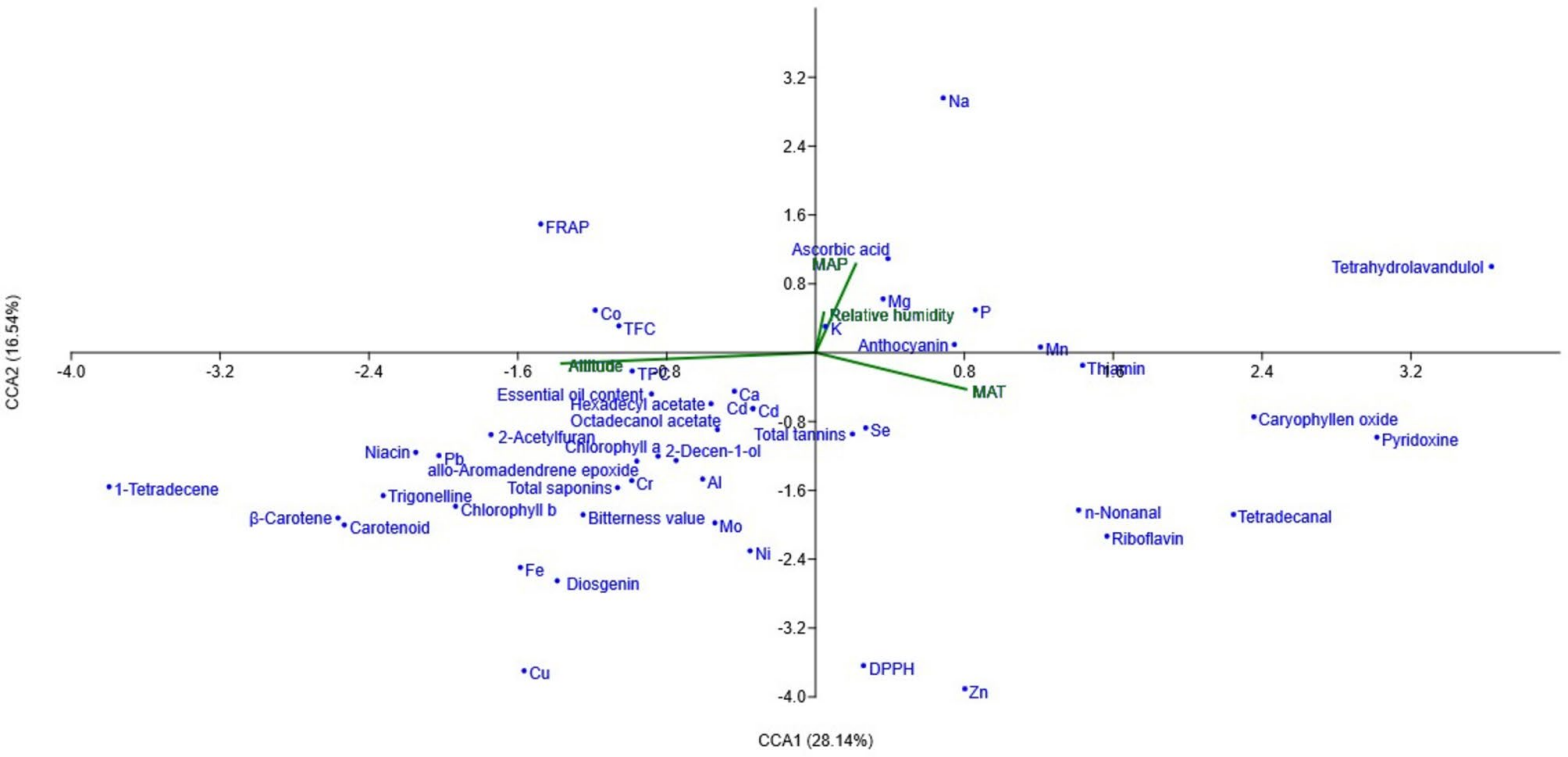
Canonical correspondence analysis (CCA) of the studied populations of *Trigonella* species collected from different environmental conditions. *MAP* mean annual precipitation, *MAT* mean annual temperature.

The studied fenugreeks populations are distributed within the latitude of 27° 15′ N to 38° 40′ N and longitude of 44° 77′ E to 60° 18′ E encompassing different geographical regions. Precipitation levels fall between 98.2 and 1892.0 mm/year, while the annual temperature averages 9.3 to 27.2 ℃. Relative humidity ranges from 28.0 to 84.2%. CCA was conducted to assess the relationship between the populations’ environmental factors and the forty-seven studied parameters of phytochemical and nutritional compounds (Fig. 10). The environmental factors that were considered included altitude, relative humidity (RH), mean annual precipitation (MAP), and mean annual temperature (MAT). The first CCA variable (CC1) concerning environmental parameters showed that MAT, MAP, and RT had a positive share, while altitude had a negative share on this CCA construction. The first canonical variable in connection to the studied characteristics showed that the value of ascorbic acid, anthocyanin, thiamin, riboflavin, pyridoxine, total tannins, and most macro-elements had a negative share in the formation of CCA1 variables. The most important factor of the second CCA (CCA2) was altitude. The majority of micro-elements, heavy metals, essential oil components, and other important traits such as trigonelline, diosgenin, and total saponins, niacin, carotenoid, TPC, TFC, and FRAP had a negative share with altitude.

The findings suggest that while environmental and climatic factors such as temperature, altitude, and precipitation do impact the variability of nutritional and phytochemical compounds in the studied fenugreeks, genotype plays the most crucial role. Interestingly, our study revealed no significant differences among the populations of the same species in terms of the most studied parameters, indicating that these factors are not solely determined by fenugreek genetic combinations and climate properties. For example, the similarity of the content of nutrients and phytochemical compounds in the populations of *T. coerulescens*, *T. filipes*, *T. calliceras*, and *T. teheranica* species can be due to the geographical and climatic origin of close distance populations.

According to the results, it can be concluded that in most cases, the differences in nutritional and phytochemical compositions among fenugreek species and populations are due to genetic factors and are less related to the geographical origin of the populations. Zhang et al.^98^ analyzed the phytochemical constituents of 747 plant species and concluded that while environmental and climatic conditions do influence the phytochemical composition of plants, the genotype is the most essential determinant, aligning with our findings. In addition, Tripodi et al.^99^, and Neugart et al.^100^ also achieved similar results. Metabolome and genome analysis can determine whether geographic patterns of variation observed in phytochemical levels are simply due to environmental flexibility or are actually due to genetic differentiation due to isolation by geographic distances.

## Conclusions

Extracts of medicinal plants and vegetables are increasingly receiving attention due to their industrial applications as natural compounds. Therefore, it is very important to identify the different masses that are the richest in terms of specialized metabolites. In this study, phytochemical diversity as well as considerable variations in vitamins and essential elements were observed among *Trigonella* species. Therefore, it is possible to improve nutritional, biochemical and phytochemical compounds by designing breeding programs and introducing new species and varieties. In summary, populations of *T. teheranica*, *T. elliptica*, *T. coerulescens*, and *T. foenum-graecum* were identified with high values of the studied traits, depending on different purposes, including functional foods as well as a therapeutic agent can be exploited.

## Data Availability

All data generated or analyzed during this study are included in this published article.
